# Multi-omics analysis of uterine fluid extracellular vesicles reveals a resemblance with endometrial tissue across the menstrual cycle: biological and translational insights

**DOI:** 10.1093/hropen/hoaf010

**Published:** 2025-02-24

**Authors:** Apostol Apostolov, Danilo Mladenović, Kadi Tilk, Andres Lõhmus, Vesselin Baev, Galina Yahubyan, Alberto Sola-Leyva, Mathilde Bergamelli, André Görgens, Cheng Zhao, Samir E L Andaloussi, Aive Kalinina, Ganesh Acharya, Fredrik Lanner, Merli Saare, Maire Peters, Paola Piomboni, Alice Luddi, Andres Salumets, Elina Aleksejeva

**Affiliations:** Celvia CC, Competence Centre on Health Technologies, Tartu, Estonia; Division of Obstetrics and Gynecology, Department of Clinical Science, Intervention and Technology (CLINTEC), Karolinska Institute, Stockholm, Sweden; Department of Gynecology and Reproductive Medicine, Karolinska University Hospital, Stockholm, Sweden; Department of Biotechnology, Institute of Molecular and Cell Biology, University of Tartu, Tartu, Estonia; HansaBioMed Life Sciences Ltd., Tallinn, Estonia; School of Natural Sciences and Health, Tallinn University, Tallinn, Estonia; Celvia CC, Competence Centre on Health Technologies, Tartu, Estonia; HansaBioMed Life Sciences Ltd., Tallinn, Estonia; Department of Molecular Biology, University of Plovdiv, Plovdiv, Bulgaria; Department of Molecular Biology, University of Plovdiv, Plovdiv, Bulgaria; Celvia CC, Competence Centre on Health Technologies, Tartu, Estonia; Division of Obstetrics and Gynecology, Department of Clinical Science, Intervention and Technology (CLINTEC), Karolinska Institute, Stockholm, Sweden; Department of Gynecology and Reproductive Medicine, Karolinska University Hospital, Stockholm, Sweden; Division of Obstetrics and Gynecology, Department of Clinical Science, Intervention and Technology (CLINTEC), Karolinska Institute, Stockholm, Sweden; Department of Gynecology and Reproductive Medicine, Karolinska University Hospital, Stockholm, Sweden; Division of Biomolecular and Cellular Medicine, Department of Laboratory Medicine, Karolinska Institutet, Stockholm, Sweden; Department of Cellular Therapy and Allogeneic Stem Cell Transplantation (CAST), Karolinska University Hospital, Huddinge, Sweden; Karolinska ATMP Center, ANA Futura, Huddinge, Sweden; Institute for Transfusion Medicine, University Hospital Essen, University of Duisburg-Essen, Essen, Germany; Division of Obstetrics and Gynecology, Department of Clinical Science, Intervention and Technology (CLINTEC), Karolinska Institute, Stockholm, Sweden; Department of Gynecology and Reproductive Medicine, Karolinska University Hospital, Stockholm, Sweden; Division of Biomolecular and Cellular Medicine, Department of Laboratory Medicine, Karolinska Institutet, Stockholm, Sweden; Department of Cellular Therapy and Allogeneic Stem Cell Transplantation (CAST), Karolinska University Hospital, Huddinge, Sweden; Karolinska ATMP Center, ANA Futura, Huddinge, Sweden; South Estonia Hospital, Võru, Estonia; Division of Obstetrics and Gynecology, Department of Clinical Science, Intervention and Technology (CLINTEC), Karolinska Institute, Stockholm, Sweden; Center for Fetal Medicine, Karolinska University Hospital, Stockholm, Sweden; Women’s Health and Perinatology Research Group, Department of Clinical Medicine, UiT—The Arctic University of Norway, Tromsø, Norway; Division of Obstetrics and Gynecology, Department of Clinical Science, Intervention and Technology (CLINTEC), Karolinska Institute, Stockholm, Sweden; Department of Gynecology and Reproductive Medicine, Karolinska University Hospital, Stockholm, Sweden; Celvia CC, Competence Centre on Health Technologies, Tartu, Estonia; Department of Obstetrics and Gynecology, Institute of Clinical Medicine, University of Tartu, Tartu, Estonia; Celvia CC, Competence Centre on Health Technologies, Tartu, Estonia; Department of Obstetrics and Gynecology, Institute of Clinical Medicine, University of Tartu, Tartu, Estonia; Department of Molecular and Developmental Medicine, University of Siena, Siena, Italy; Department of Molecular and Developmental Medicine, University of Siena, Siena, Italy; Celvia CC, Competence Centre on Health Technologies, Tartu, Estonia; Division of Obstetrics and Gynecology, Department of Clinical Science, Intervention and Technology (CLINTEC), Karolinska Institute, Stockholm, Sweden; Department of Gynecology and Reproductive Medicine, Karolinska University Hospital, Stockholm, Sweden; Department of Obstetrics and Gynecology, Institute of Clinical Medicine, University of Tartu, Tartu, Estonia; Celvia CC, Competence Centre on Health Technologies, Tartu, Estonia; Department of Obstetrics and Gynecology, Institute of Clinical Medicine, University of Tartu, Tartu, Estonia

**Keywords:** endometrium, uterine fluid, extracellular vesicles, endometrial receptivity, implantation, endometrial organoids

## Abstract

**STUDY QUESTION:**

Does the molecular composition of uterine fluid extracellular vesicles (UF-EVs) reflect endometrial tissue changes across the menstrual cycle?

**SUMMARY ANSWER:**

Concordance between endometrial tissue and UF-EVs exists on miRNA and mRNA levels along the menstrual cycle phases and UF-EV surface proteomic signatures suggest EVs originate from several major endometrial cell populations.

**WHAT IS KNOWN ALREADY:**

The clinical value of endometrial receptivity testing is restricted by invasiveness and the use of only one omics level of input. There is promising evidence that UF-EVs can reflect changes in mid-secretory endometrium, highlighting the potential to establish endometrial receptivity testing right before embryo transfer. However, the dynamic changes of UF-EVs molecular cargo have not been directly compared to endometrial tissue on multiple omics levels.

**STUDY DESIGN, SIZE, DURATION:**

This cross-sectional study included fertile women from four menstrual cycle phases: proliferative and early-, mid-, and late-secretory phases. In total, 26 paired samples of UF and endometrial tissue were collected. mRNA and miRNA were sequenced, and differential analysis was performed on consecutive phases. UF-EVs were profiled for various protein surface markers associated with different cell types. EVs from epithelial endometrial organoid-conditioned culture media were used as a reference of pure epithelial endometrial EVs.

**PARTICIPANTS/MATERIALS, SETTING, METHODS:**

Paired UF and endometrial tissue samples were collected from 26 fertile, reproductive-age women. EV isolation from UF was validated using electron microscopy and western blotting, and particle numbers were measured by nanoparticle tracking analysis. The transcriptome and miRNome of UF-EVs and endometrial tissue were sequenced, and differential expression analysis was conducted on consecutive phases of the menstrual cycle. Bead-based EV flow cytometry targeting 37 surface protein markers was used to characterize EVs from UF and endometrial organoids.

**MAIN RESULTS AND THE ROLE OF CHANCE:**

Surface proteome analysis revealed that UF-EVs from the mid-secretory phase had significantly increased expression of natural killer cell marker CD56 (*P* < 0.005), pan-leukocyte marker CD45 (*P* < 0.005), pan-T-cell marker CD3 (*P* < 0.005), and coagulation-related protein CD142 (*P* < 0.005) compared to those from the proliferative phase, whereas markers associated with endometrial epithelial cells (CD29, CD133, and CD326) did not significantly change across the menstrual cycle. Transcriptomic analysis highlighted differential expression of histone and metallothionein genes that correlated between paired UF-EVs and endometrial tissues in each tested menstrual cycle phase. Principal component analysis of miRNomes of paired UF-EVs and endometrial tissue samples resulted in similar clustering patterns, where mid- and late-secretory samples clustered closely, and proliferative and early-secretory phase samples clustered separately. Half of the differentially expressed miRNAs in each phase in UF-EVs were also differentially expressed in the endometrium. Importantly, nine mid-secretory phase UF-EV DE miRNAs were identified, five of which were common between UF-EVs and endometrial biopsies, including hsa-miR-30d-5p and hsa-miR-200b-3p, both of which were previously implicated in implantation. Notably, three of the nine miRNAs, hsa-miR-200b-3p, hsa-miR-141-3p, and hsa-miR-200a-3p, were predicted to regulate mRNAs in the endometrial tissue and the pre-implantation embryo trophectoderm.

**LARGE SCALE DATA:**

N/A

**LIMITATIONS, REASONS FOR CAUTION:**

The clinical dating of the menstrual cycle phase is based on the first day of menstruation and the time of the LH peak, which does not exclude the possibility that the expected endometrial phase was not reached. The wider limitation of our study is the lack of standardized procedures for collecting UF samples in gynaecological practice, which could challenge the replication of our findings.

**WIDER IMPLICATIONS OF THE FINDINGS:**

Evidence that UF-EVs reflect endometrial phases of menstrual cycle supports the use of UF-EVs in endometrial receptivity testing. Additionally, further studies of UF-EVs in endometrial pathologies could be beneficial for diagnostics, considering that more invasive tissue biopsies only reflect the biopsy site and not the full endometrium.

**STUDY FUNDING/COMPETING INTEREST(S):**

This study was supported by the European Regional Development Fund Enterprise Estonia’s Applied Research Program under the grant agreement number 2014-2020.4.02.21-0398 (EVREM), the Estonian Research Council (grant nos. PRG1076 and PSG1082), the Horizon Europe NESTOR grant (grant no. 101120075) of the European Commission, the Swedish Research Council (grant no. 2024-02530), the Novo Nordisk Fonden (grant no. NNF24OC0092384), and the National Recovery and Resilience Plan of the Republic of Bulgaria, project number BG-RRP-2.004-0001-C01. A.S.L. received funding from the Becas Fundación Ramón Areces para Estudios Postdoctorales. All the authors declare no conflict of interest.

WHAT DOES THIS MEAN FOR PATIENTS? The inner lining of the uterus, i.e. the endometrium, undergoes complex changes each month to reach a state that is ready for embryo implantation. This optimal time when the endometrium becomes receptive and ready for embryo implantation, known as the ‘window of implantation’, varies among women. To ensure that an embryo is transferred at the right time in assisted reproduction treatments, clinicians can take an endometrial tissue sample and assess it. As the endometrial biopsy cannot be taken right before embryo transfer, the variability across the menstrual cycle reduces the diagnostic accuracy of the tissue assessment. In our study, we have shown that instead of using invasive tissue biopsies, the endometrium can be assessed more easily by small vesicles that are secreted into uterine fluid. Additionally, endometrial tissue is complex, and the assessment of its state could profit from combining different types of molecules. We demonstrated that different types of molecules carried by the uterine fluid vesicles can be used as biomarkers. Our research forms the basis of the development of a new diagnostic test that could accelerate the time to pregnancy and thereby reduce the emotional, physical, and financial burdens of multiple assisted reproduction cycles.

## Introduction

Considering the intricate changes occurring in the endometrium each cycle to reach a receptive state, its clinical testing has perplexingly limited benefit in subfertile patients (Arian *et al.*, 2023; Glujovsky *et al.*, 2023). Even if a shift in the window of implantation (WOI) can be detected, the requirement of a tissue biopsy to verify receptivity status is a significant limitation as it cannot be performed right before the embryo transfer. Aspiration of uterine fluid (UF) before embryo transfer does not affect the implantation rates; therefore, UF is a promising way to assess endometrial receptivity in a minimally invasive way (van der Gaast *et al.*, 2003; Hou *et al.*, 2022; Saraee *et al.*, 2022).

UF contains extracellular vesicles (EVs) that are secreted by cells either through plasma membrane budding or an endosomal system (Aleksejeva *et al.*, 2022). Interestingly, a higher production of EVs in mid-secretory endometrium has been observed in mice, and there is evidence that this occurs in a similar way in humans (Ng *et al.*, 2013; Vilella *et al.*, 2015; Tan *et al.*, 2020). There is increasing evidence that EVs mediate maternal–embryonic communication by facilitating the transfer of molecules from the maternal side to the embryo and vice versa (Vilella *et al.*, 2015; Liu *et al.*, 2020; Makieva *et al.*, 2024). Importantly for diagnostic purposes, EVs contain mRNA, miRNA, proteins, and metabolites reflective of the physiological state of the cell or tissue of origin (Larssen *et al.*, 2017; Dwivedi *et al.*, 2023; Gokulnath *et al.*, 2024). Additionally, the pre-enrichment of EVs improves biomarker molecule detection sensitivity compared to bulk extraction of molecules (Ibañez-Perez *et al.*, 2022). We have recently demonstrated, using 68 tissue-based mRNA biomarkers of endometrial receptivity, that the UF-EV transcriptome allows us to differentiate receptive from pre-receptive endometrium, although with some inaccuracies (Meltsov *et al.*, 2023a). Other groups have reported implantation-related miRNA, mRNAs, and protein cargo in UF-EVs; however, they have not explored whether UF-EV cargoes mirror the changes in endometrial tissue, nor have they explored the changes throughout the menstrual cycle (Giacomini *et al.*, 2021; Li *et al.*, 2021; Rai *et al.*, 2021; Ibañez-Perez *et al.*, 2022). Therefore, an integrated systematic profiling of the UF-EVs and endometrial tissue across the menstrual cycle was needed to provide deeper insight into the biological processes that regulate endometrial maturation and how that can be translated into diagnostics and therapeutics. Furthermore, incorporating biomarkers from several omics levels could improve the accuracy of endometrial receptivity testing, as has been shown for other diseases (Sturm *et al.*, 2023).

In this study, we aimed to elucidate the changes in the miRNome, transcriptome, and surface proteome of UF-EVs along the menstrual cycle in healthy fertile women. Furthermore, we profiled the transcriptome and miRNome of paired endometrial biopsies (EB) to compare the dynamic changes in tissue and UF-EVs. As a reference, we generated endometrial epithelial organoids (EEOs) from fertile women and characterized their EVs (EEO-EVs).

## Materials and methods

### Ethical approval

The study was approved by the Research Ethics Committee of the University of Tartu, Estonia (No. 330M-8), and written informed consent was obtained from all participants.

### Participants and sample collection

Healthy and fertile women of reproductive age were recruited from South Estonian Hospital (Võru, Estonia). The inclusion criteria required participants to have at least one live-born child and to avoid hormonal medications for at least 3 months prior to the study. Participants were excluded if they had sexually transmitted diseases, uterine pathologies, endometriosis, or polycystic ovary syndrome. Paired EB (n = 26) and UF (n = 26) samples were obtained at four menstrual cycle phases: proliferative (P, n = 5), early-secretory (ES, n = 7), mid-secretory (MS, n = 7) and late-secretory phase (LS, n = 7). P phase was determined using menstrual cycle history. ES, MS, and LS phases were determined by counting the days after the LH peak as detected with a BabyTime hLH urine cassette (Pharmanova, Belgrade, Serbia). Specifically, we designated LH + 2 and LH + 3 as ES, LH + 7 to LH + 9 as MS, and LH + 11 to LH + 12 as LS. UF samples were obtained through transcervical lavage with 0.5 ml of phosphate-buffered saline (PBS) using a sterile intrauterine insemination catheter (Cooper Surgical, Trumbull, CT, USA), ensuring minimal insertion to avoid uterine fundus contact. After the procedure, the catheter and UF were examined visually to verify that no tissue was accidentally collected. UF was centrifuged for 5 min at 500*g*, and the supernatant was kept at −80°C. EB samples were collected using a Pipelle flexible suction catheter (Laboratoire CCD, Paris, France) and placed in HypoThermosol FRS Preservation Solution (Sigma, St. Louis, MO, USA). EB samples were divided for endometrial organoid generation and for transcriptomic analysis stored in RNAlater (Thermo Fisher Scientific, Waltham, MA, USA).

### Endometrial organoid culture

The epithelial glands were extracted from four fresh EB (two from P and MS phases) by tissue dissociation, and EEOs were generated and cultivated as previously described (Turco *et al*., 2017). For the experiment, the formed EEOs were dissociated into single cells, and 15 000 cells were seeded in 25 µl of Matrigel (Corning, Corning, NY, USA) on 48-well plates; each condition had 24 replicates. Once organoids were expanded, we proceeded with hormonal stimulation to recapitulate menstrual phases; 10 nM β-estradiol (E2, Sigma) treatment lasted for 48 h, followed by either (i) 10 nM E2 or (ii) a combination of 10 nM E2, 1 µM progesterone (P4, Sigma), and 1 µM 8-bromoadenosine 3′,5′-cyclic monophosphate (cAMP, Sigma) for 4 days. For controls, organoids maintained in expansion media were used.

### Extracellular vesicle isolation

EVs were isolated from UF using miniPURE-EV SEC columns (HansaBioMed Life Sciences, Tallinn, Estonia). Each fraction was measured with nanoparticle tracking analysis (NTA), and fractions 9–15 containing the EVs were pooled. For the isolation of EVs from EEOs, 6 ml of media were concentrated down to 2 ml using Amicon Ultra-4 100 kDa centrifugal filters (Merck, Darmstadt, Germany) and then purified using PURE-EV SEC columns (HansaBioMed Life Sciences). Each resulting fraction was analyzed by NTA, and fractions 6–11 were subsequently pooled. The pooled EV suspension was directly used for multiplex bead-based EV flow cytometry. For EV-RNA isolation, EVs were precipitated from the EV suspension with the evGAG (HansaBioMed Life Sciences), using reagent-to-sample volume ratio of 1:1, according to the manufacturer’s instructions.

### Nanoparticle tracking analysis

NTA was performed using a ZetaView PMX-120 instrument (Particle Metrix, Inning am Ammersee, Germany) running on software version 8.05.12 SP1. All measurements were performed with the same camera sensitivity and shutter speed. FCS files were generated from each measurement, and Floreada online software (https://floreada.io/) was used to calculate the median and mean particle size for all individual samples.

### Western blots

Western blot (WB) was used to verify the presence of commonly used EV intraluminal markers ALIX and TSG101 in isolated UF-EVs (Welsh *et al.*, 2024). evGAG-precipitated EV pellets were combined with Laemmli buffer and β-mercaptoethanol at a ratio of 1:5. Samples were heated at 95°C for 5 min and loaded onto a 7.5% polyacrylamide gel. Electrophoresis was conducted at room temperature (RT), starting with 40 V for 15 min, followed by 60 V for 1.5 h. Wet transfer onto a nitrocellulose membrane was performed for 1 h at 0°C and 350 mA. Post-transfer, Ponceau S staining was used to assess total protein (Thermo Fisher Scientific). The membranes were blocked with 3% skimmed milk in PBS–Tween 20 (PBS-T) (0.1%) at RT for 1 h, and incubated overnight at +4°C on a shaker with the primary antibodies mouse anti-ALIX (HansaBioMed Life Sciences) and mouse anti-TSG101 (HansaBioMed Life Sciences), each diluted at 1:500 in 3% skimmed milk in PBS-T. Subsequently, the membranes were washed with PBS-T for 15 min and incubated at RT with goat anti-mouse HRP-conjugated secondary antibodies (HansaBioMed Life Sciences) diluted 1:5000 in PBS-T without milk. After washing, signal detection was performed using SuperSignal™ West Pico PLUS Chemiluminescent Substrate (Thermo Fisher Scientific) as per the manufacturer’s instructions. Imaging was conducted using the G:BOX gel documentation system (Syngene, Bangalore, India).

### EV profiling by multiplex bead-based EV flow cytometry

For phenotyping of the protein surfaceome of UF-EVs and EEO-EVs, the surface proteome flow cytometry-based analysis Human IO EV Kit (Miltenyi Biotec GmbH, Bergisch Gladbach, Germany) was used. Median fluorescence intensity (MFI) of each marker was adjusted by subtracting the MFI of the blank control utilized in the same experimental run. UF-EV samples from P (n = 4), ES (n = 6), MS (n = 5), and LS (n = 5) were used. The EEO-EVs (n = 4) from three different conditions (untreated, E2 treated, and E2 + P4 + cAMP treated) were used. The sample input was normalized by nanoparticle counts (1 × 10^9^ per sample), as described previously (Wiklander *et al.*, 2018; Nguyen *et al.*, 2024). The heatmap to visualize the results was created with Morpheus (https://software.broadinstitute.org/morpheus), and the box plots were created with GraphPad Prism 10.2.3 (Boston, MA, USA).

### Transmission electron microscopy

EVs were prepared for transmission electron microscopy (TEM) as described previously (Puhka *et al.*, 2017). Briefly, EVs were fixed with 2.0% paraformaldehyde (PFA) fixation in NaPO_4_ buffer and stained with 2% neutral uranyl acetate to enhance contrast and visualize their internal structures. Then, EVs were embedded in a mixture of uranyl acetate and methyl cellulose (1.8/0.4%). A volume of 30 µl from each EV SEC suspension (∼1 e11 p/ml) was used directly (EEO-EVs) or after precipitation with evGAG (UF-EVs). Hitachi HT7800 TEM operating at 100 kV was used for TEM imaging. High-resolution images were acquired with a Gatan Rio9 bottom-mounted CMOS camera (model 1809, Gatan Inc., Pleasanton, CA, USA).

### RNA extraction, library preparation, and sequencing

RNA from EBs, UF-EVs, and EEO-EVs was extracted with QIAzol (Qiagen, Germany) and miRNeasy micro kits (Qiagen, Hilden, Germany) by consecutively separating the large and small RNA fractions. RNA quality and quantity were checked on a Bioanalyzer TapeStation 2100 (Agilent, Santa Clara, CA, USA) with High Sensitivity RNA ScreenTape (Agilent) and RNA ScreenTape (Agilent) for UF-EVs and EBs, respectively. The small RNA fraction was quantified with a Qubit microRNA assay (Thermo Fisher Scientific). The small RNA libraries were generated using a NEXTflex small RNA library preparation kit v4 with 20 ng of each sample used as the starting concentration (PerkinElmer, Waltham, MA, USA). mRNA libraries were performed utilizing the TruSeq exome RNA library preparation kit (Illumina, San Diego, CA, USA) which enriches for coding regions with sequence-specific probes and does not depend on a poly(A) tail. We used 100 ng of RNA from EB samples and 100 ng, or the maximum quantity of RNA, from UF-EV samples. The completed libraries were pooled and then evaluated using High Sensitivity DNA ScreenTape D1000 (Agilent); 1 nM library was sequenced using the NextSeq 1000 platform (Illumina) at a single-end 80 bp read setting.

### Preprocessing, mapping, and quality control

The raw reads were pre-processed using FastQC and Trim Galore. The identification and categorization of microRNAs were performed with the miRGalaxy platform (Glogovitis *et al.*, 2021). The small RNA libraries were generated from 4 to 14 million reads. The proportion of mapping rate ranged from 69% to 95% with miRNAs. The range of reads in the RNA exome sequencing across the samples varied from 5 to 64 million; only four samples fell below 10 million, with the average around 20 million reads. A significant proportion of these reads, between 85% and 96%, successfully aligned to the human genome version 38 (GRCh38) using HITSAT2, and over 90% of these alignments were to exonic regions.

### miRNA differential expression analysis

Differentially expressed miRNAs (DEmiRs) were identified using the DESeq2 package (Love *et al.*, 2014). miRNAs identified with fold change |log2FC| ≥ 1 and a *P*_adj_ < 0.05 were selected for downstream analyses (Tang *et al.*, 2023).

### Comparative transcript analysis

Differential expression analysis was done with DeSeq2 (Love *et al.*, 2014) and differentially expressed genes (DEGs) were identified with filters baseMean >15, |log2FC| ≥ 1 and a *P*_adj_ < 0.05 were selected for subsequent analysis. Gene ontology (GO) enrichment analysis was conducted using g:Profiler (Kolberg *et al.*, 2023), and the chord plot was created with SRplot (Tang *et al.*, 2023).

### Construction of the miRNA–mRNA network

The miRNA-mRNA interaction network was constructed using miRTarVis+ (L'Yi *et al.*, 2017) for DE menstrual cycle-specific UF-EVs miRNAs and DE endometrial putative target mRNAs. The analysis targeted opposite direction interactions between miRNAs and mRNAs with applied filter *P*_adj_ < 0.05 and fold change > 1.2. Only the predicted interactions confirmed by two databases, TargetScan and microRNA.org (integrated within miRTarVis+), were considered. In UF-EV miRNA and human embryo mRNA network analysis, we used TargetScan (McGeary *et al.*, 2019) for miRNA target prediction, and the clusters of trophectoderm (TE)-trajectory-related transcription factor (TF) genes, along with their expression data and annotation of embryonic cells, were extracted from our recent publication (Zhao *et al.*, 2025).

### Statistical analyses

Statistical analyses were performed using GraphPad Prism 10.2.3 (La Jolla, CA, USA). Multiplex bead-based EV flow cytometry data were analyzed using Welch’s *t*-test (a two-tailed *t*-test for independent samples with unequal variances), followed by a Bonferroni correction to assess the robustness of the significance. After the correction, *P*-values <0.017 were considered significant. One-way ANOVA was conducted to compare the median size of UF-EVs measured by NTA. Pearson correlation analysis on mRNA expression values between paired UF-EVs and endometrial tissues was performed with SRplot (Tang *et al.*, 2023).

## Results

### Basic characterization of UF-EVs across the menstrual cycle

**Figure 1.**
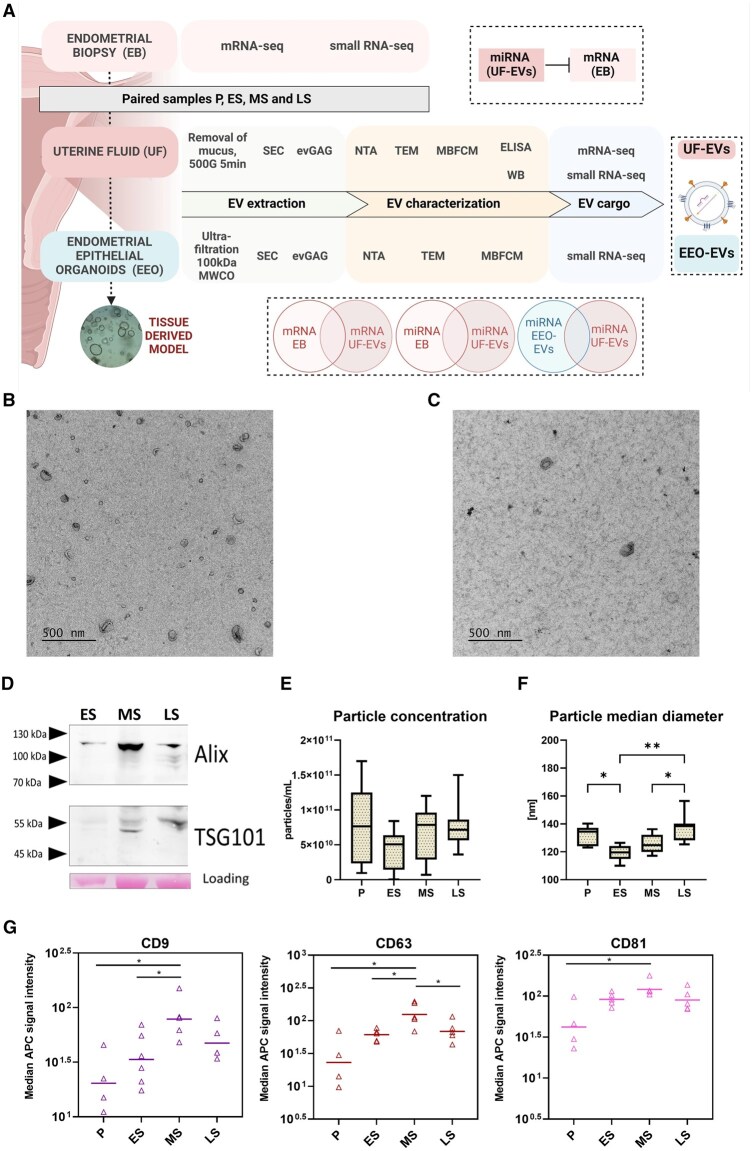
**Basic characterization of uterine fluid-derived extracellular vesicles (UF-EVs).** (**A**) Workflow of paired UF-EV, endometrial biopsy (EB), and endometrial epithelial organoid EV (EEO-EV) isolation and further analysis. Representative TEM image of (**B**) UF-EVs and (**C**) EEO-EVs. (**D**) Western blot of ALIX and TSG101 in EVs isolated from UF using SEC and evGAG. (**E**) Particle concentration (with minimum to maximum and interquartile range) of UF-EVs across the menstrual cycle measured by NTA. (**F**) Median particle diameter (with minimum to maximum and interquartile range) of UF-EVs across the menstrual cycle measured by NTA. (**G**) Expression dynamics of EV surface proteins CD9, CD63, and CD81 in UF-EVs across the menstrual cycle measured by multiplex bead-based EV flow cytometry (MBFCM). Equal amounts of particles were taken from each sample for analysis. Statistical significance for the flow cytometry results was determined by Welch’s *t*-test and for NTA results by one-way ANOVA with Tukey’s multiple comparisons test (*P < 0.05, **P < 0.005). P, proliferative; ES, early-secretory; MS, mid-secretory; LS, late-secretory; MWCO, molecular-weight cutoff SEC, size-exclusion chromatography; MBFCM, multiplex bead-based EV flow cytometry; evGAG, glycosaminoglycan precipitation; TEM, transmission electron microscopy; NTA, nanoparticle-tracking analysis; MFI APC, median fluorescence intensity of allophycocyanin.

We isolated UF-EVs from fertile women (n = 26) from P, ES, MS and LS phases using SEC as it depletes soluble proteins and ribonucleocomplexes and enables multiple analyses from a single sample (Ibañez-Perez *et al.*, 2022; Kim *et al.*, 2023) (Fig. 1A). After isolating EVs with SEC, evGAG was used to precipitate the EVs for downstream WB, TEM, and RNA analyses. TEM images captured EVs with typical morphology and size in EV isolates from UF (Fig. 1B) and EEO media (Fig. 1C). WB analysis confirmed the presence of commonly used EV intraluminal markers, ALIX and TSG101, in UF-EVs (Fig. 1D). Next, we assessed the number and size of particles in UF-EVs samples using NTA. Particle concentrations were variable, likely due to technical variability during uterine lavage recovery from women (Fig. 1E). The median diameter of UF-EVs across the menstrual cycle was 126.8 ± 10.1 nm. We saw a significant decrease in the median diameter of particles in ES compared to P (119.6 ± 5.7 nm vs 134.5 ± 6.9 nm, *P* < 0.05) and MS compared to LS (126.7 ± 6.8 nm vs 138.8 ± 9.9 nm, *P* < 0.05) phases (Fig. 1F). It is important to note that UF is a complex biological fluid that may also contain lipoproteins. These lipoproteins are detected as particles by NTA but are not visualized using the TEM protocol employed in our study. As a result, the number of particles observed in the TEM image is expected to be lower than those detected by NTA. To estimate the proportion of EVs among nanoparticles, we took equal amounts of particles across phases and measured EV surface proteins (tetraspanins CD9, CD63, CD81) using multiplex bead-based EV flow cytometry. There was a significant enrichment of all tetraspanins in the MS phase respective to the P phase, and additionally, a significant enrichment of CD63 particles in the MS phase respective to the LS phase, suggesting there is an increased secretion of EVs during the WOI. However, after Bonferroni correction, CD9 (*P* = 0.017), CD63 (*P* = 0.018), and CD81 (*P* = 0.05) were no longer significant (Fig. 1G).

### The source of UF-EVs extends beyond the endometrial epithelium

**Figure 2.**
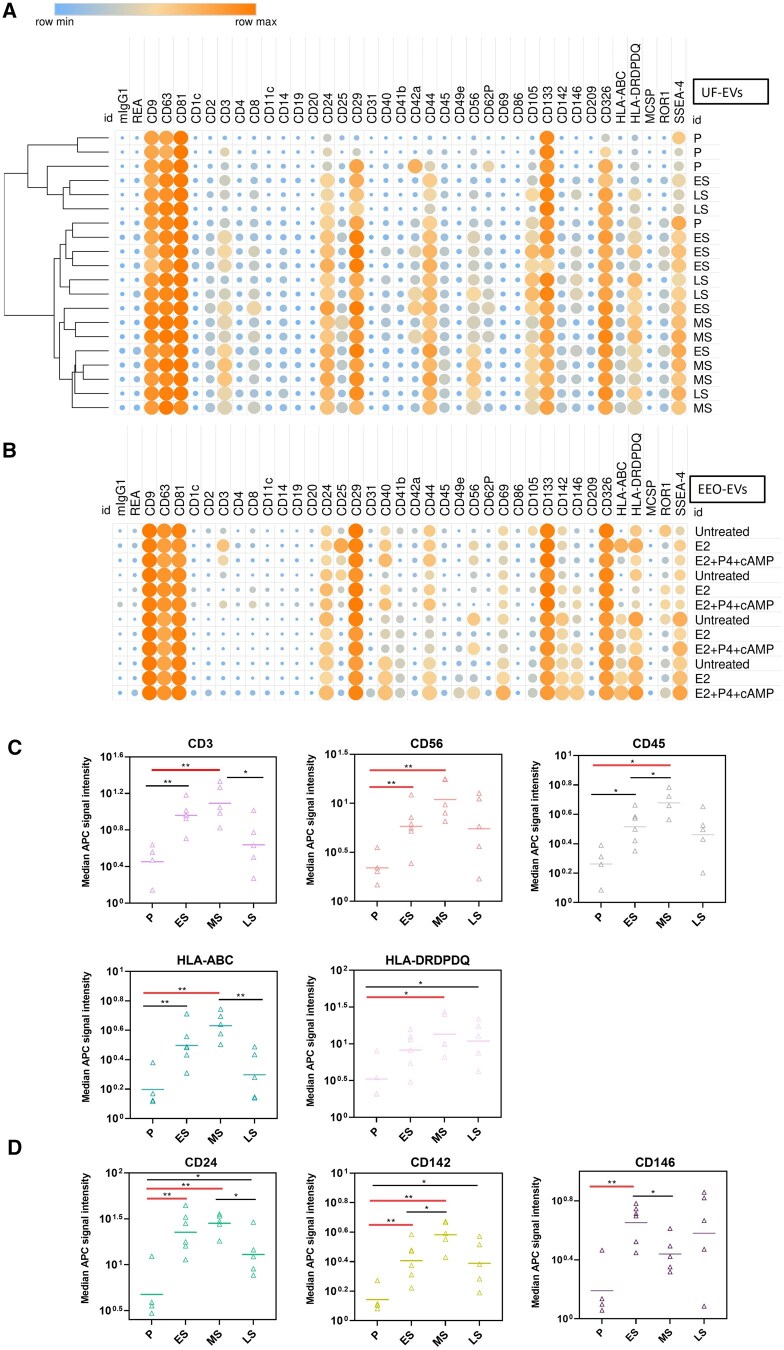
**Surface proteome signature of uterine fluid extracellular vesicles (UF-EVs) and endometrial organoid EVs (EEO-EVs).** Heatmap showing the surface proteome signature of (**A**) UF-EVs across menstrual cycle phases and (**B**) EEO-EVs from untreated and hormonally treated organoids. (**C**) Immunity-related protein expression on the surface of UF-EVs. (**D**) Angiogenesis, coagulation, and adhesion marker expression in UF-EVs. Equal amounts of particles were taken from each sample for analysis. Statistical significance was determined by Welch’s *t*-test (*P < 0.05, **P < 0.005). Bonferroni-corrected significant values are depicted with red line. P, proliferative; ES, early-secretory; MS, mid-secretory; LS, late-secretory; E2, estradiol; P4, progesterone; cAMP, cyclic adenosine monophosphate; MFI APC, median fluorescence intensity of allophycocyanin.

To assess EV heterogeneity and their potential cell-of-origin in UFs, we performed multiplex bead-based flow cytometric analysis of 37 surface proteins, which includes the three EV tetraspanin markers reported in Fig. 1G. As a reference, we characterized the surfaceome of endometrial epithelial EVs by analyzing EEO-EVs from organoids treated with E2 (corresponding to the ES phase) and a mixture of E2, P4, and cAMP (corresponding to the MS phase). Comparison of UF-EVs to EEO-EVs indicates that CD29, CD133, CD326/EpCAM, CD24, CD40, CD44, CD142/Tissue factor, CD146, HLA-DR/DP/DQ, ROR1, and SSEA-4 signals in UF-EVs originate from epithelial cells (Fig. 2A and B). Unexpectedly, T-cell and natural killer (NK) cell markers (CD3 and CD56, respectively) were detected on EVs from some EEOs; however, the signals were near background level and 5-fold lower than in UF-EVs, indicating that CD3 and CD56 positive EVs in UF likely originate from immune cells. Although hierarchical clustering analysis of UF-EVs using these 37 markers did not group the samples according to the menstrual cycle phases (Fig. 2A), we saw significant changes in individual markers: CD3 (pan-T-cell), HLA-DR/DP/DQ (pan-antigen-presenting cell), CD56 (uterine natural killer [uNK] cells), CD45 (pan-leukocyte) and HLA-A/B/C (all cells) were significantly increased in the MS phase compared to the P phase (Fig. 2C). Expression of markers associated with adhesion (CD24 and CD146/MUC18) and coagulation (CD142/Tissue Factor) also significantly changed (Fig. 2D).

Next, we compared the profiles of miRNAs in UF-EVs and corresponding hormonally treated EEO-EVs. We only considered miRNAs that had at least 50 counts per million in our samples. UF-EVs in the ES phase and E2-treated EEO-EVs had 191 common miRNAs; however, there were 139 miRNAs exclusively in UF-EVs and 21 miRNAs in EEO-EVs (Supplementary Fig. S1A). UF-EVs in the MS phase and EEO-EVs treated with E2 + P4 + cAMP had 187 common miRNAs and 141 exclusive miRNAs in UF-EVs and 14 miRNAs only in EEO-EVs (Supplementary Fig. S1B). This shows that the UF-EV miRNome is more complex than that of EEO-EVs, implying that UF-EVs are secreted from more diverse cellular sources than just endometrial epithelial cells. Collectively these findings suggest that UF-EVs originate from several cell types, including endometrial epithelial cells, endothelial cells, T cells, and NK cells.

### Transcriptomic changes in UF-EVs and paired EBs along the menstrual cycle

**Figure 3.**
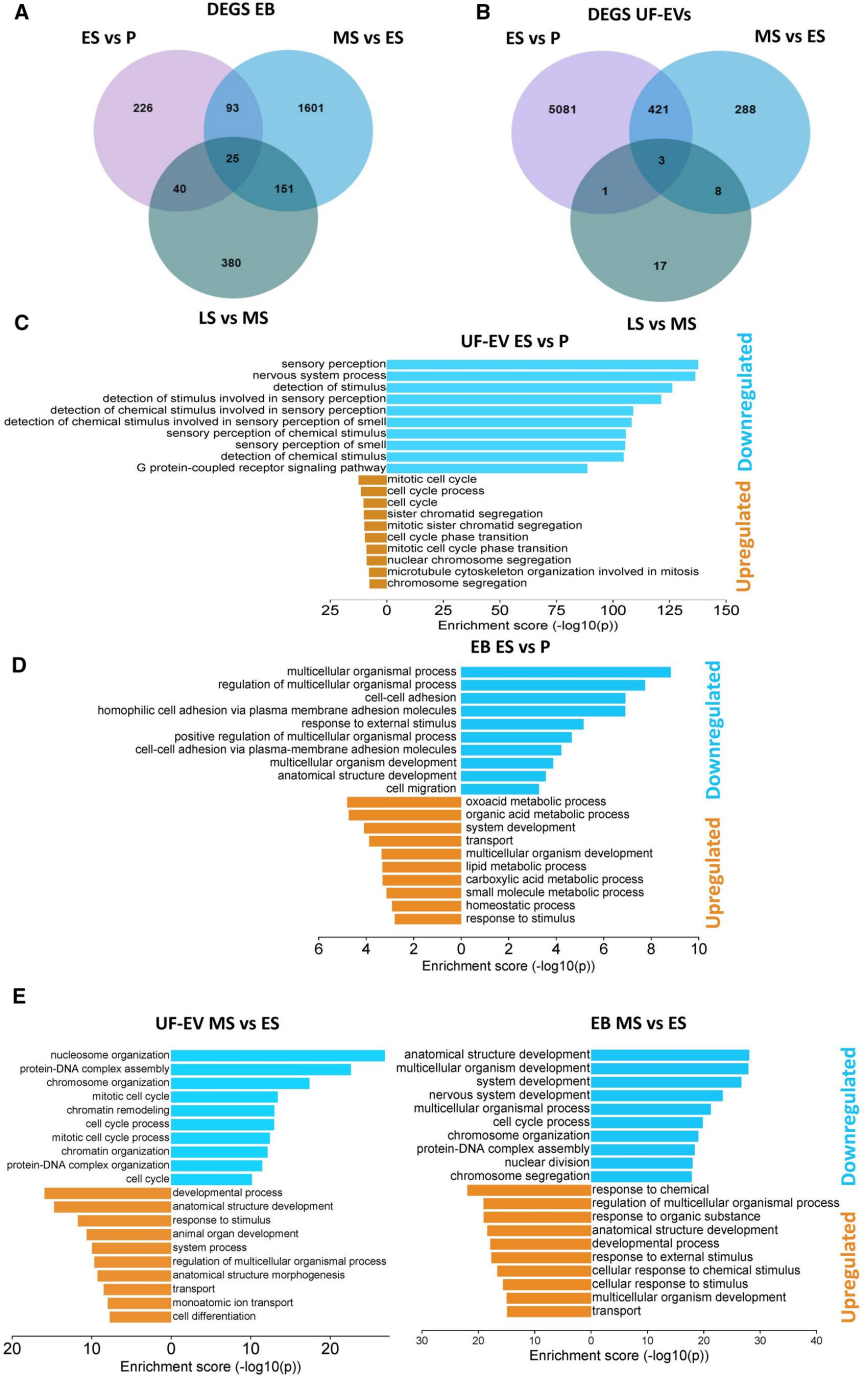
**Transcriptomic changes in endometrial tissue biopsies (EB) and uterine fluid extracellular vesicles (UF-EVs) across the menstrual cycle.** (**A**) Numbers of DEGs during menstrual cycle transitions in endometrial tissue (EB). (**B**) Numbers of DEGs during menstrual cycle transitions in UF-EVs. (**C**) Gene ontology analysis of the upregulated and downregulated biological pathways in ES compared to P phase in UF-EVs. (**D**) Gene ontology analysis of the upregulated and downregulated biological pathways in ES compared to P phase in EBs. (**E**) Gene ontology analysis of the upregulated and downregulated biological pathways in MS compared to ES phase in UF-EVs and EBs. DEGs, differentially expressed genes; P, proliferative; ES, early-secretory; MS, mid-secretory; LS, late-secretory phases.

To identify the DEGs along the menstrual cycle, RNA-seq of UF-EVs and paired EBs was performed (P, n = 5 + 5; ES, n = 7 + 7; MS, n = 7 + 7; and LS, n = 7 + 7). Gene expression levels were analyzed by comparing each phase of the menstrual cycle with the preceding phase to identify consecutive changes. In endometrial tissue, we observed the biggest change in the MS compared to the ES phase (959 upregulated and 911 downregulated DEGs) (Fig. 3A, Supplementary Table S1), whereas in UF-EVs the most dramatic change occurred earlier, in the ES compared to the P phase (5212 downregulated and 294 upregulated DEGs) (Fig. 3B, Supplementary Table S2). Compared to endometrial tissue, the changes in UF-EVs in the LS phase were modest (596 vs 29 DEGs, respectively).

Subsequently, we assessed whether the UF-EV transcriptome reflects the biological processes that occur in the tissue during the menstrual cycle. Based on GO analysis, UF-EVs do not reflect biological processes occurring in endometrial tissue in the ES compared to the P phase (Fig. 3C). However, in the MS compared to the ES phase, similar changes occur in the tissue and UF-EVs: cell cycle and chromosome-related processes are downregulated, whereas response to stimulus, development, and transport are upregulated (Fig. 3D). The comparison of UF-EV transcriptome from the LS to the MS phase did not reveal many DEGs (Fig. 3B), and the corresponding biological process did not match with those of the EB (Supplementary Fig. S2).

**Figure 4.**
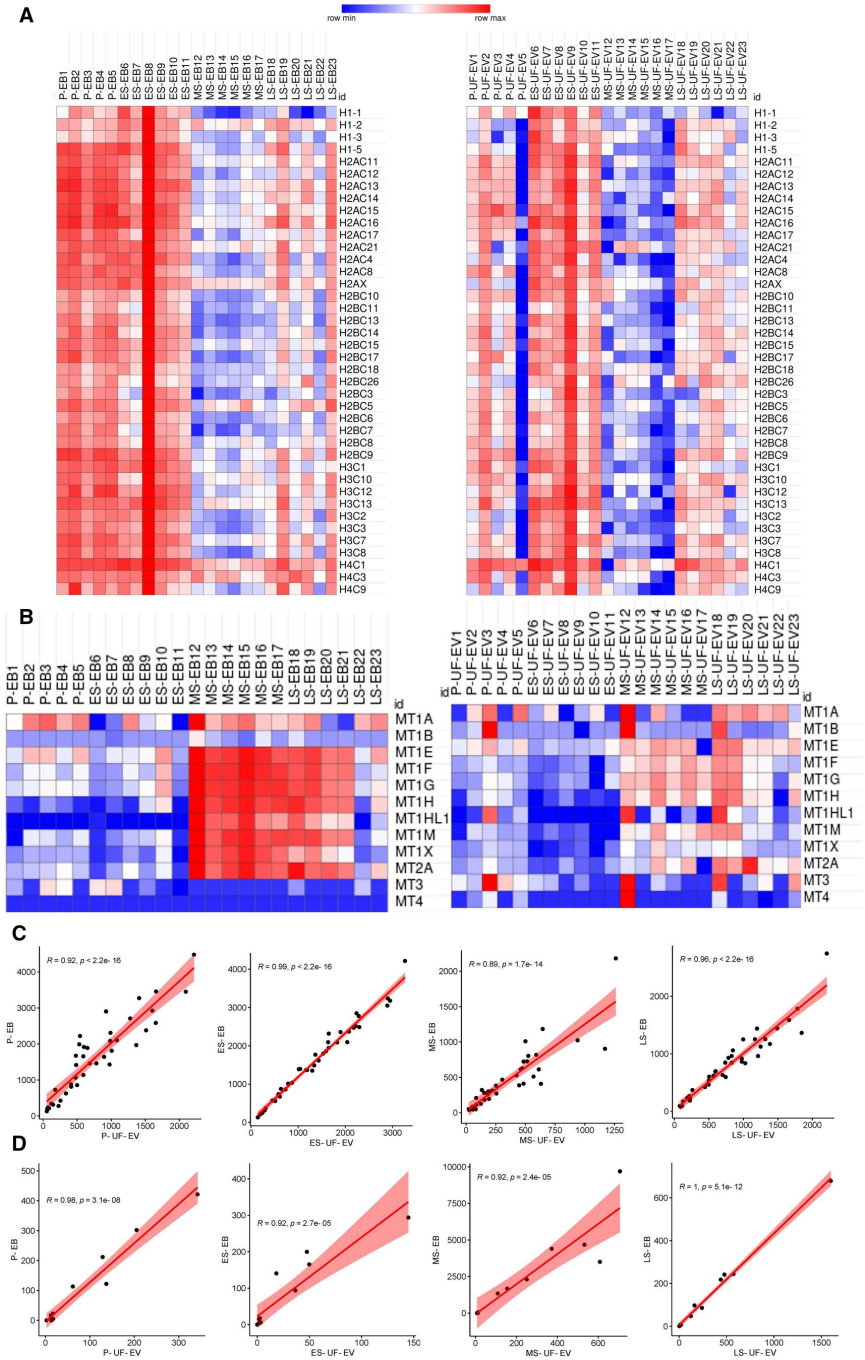
**Histone and metallothionein family gene expression changes in endometrial tissues (EB) and paired uterine fluid extracellular vesicles (UF-EVs) across the menstrual cycle.** Heatmaps of normalized gene expression levels of (**A**) histones and (**B**) metallothioneins in paired EB (on the left) and UF-EV (on the right) samples. (**C**) Pearson correlation of the expression of histones between UF-EVs and EBs in each phase of the menstrual cycle. (**D**) Pearson correlation of the expression of metallothioneins between UF-EVs and EBs in each phase of the menstrual cycle. P, proliferative; ES, early-secretory; MS, mid-secretory; LS, late-secretory.

Among the DEGs, many histones and metallothioneins were identified (Supplementary Tables S1 and S2). We focused on these two large families to compare the dynamics of gene expression changes in EBs and paired UF-EV samples (Fig. 4A and B). The Pearson correlation coefficient for histones and metallothioneins expression between EB and UF-EV is close to 1 in each menstrual phase, indicating a strong positive linear relationship (Fig. 4C and D). The expression of metallothioneins is downregulated during the P and ES phases, whereas it is upregulated during the MS and LS phases.

**Figure 5.**
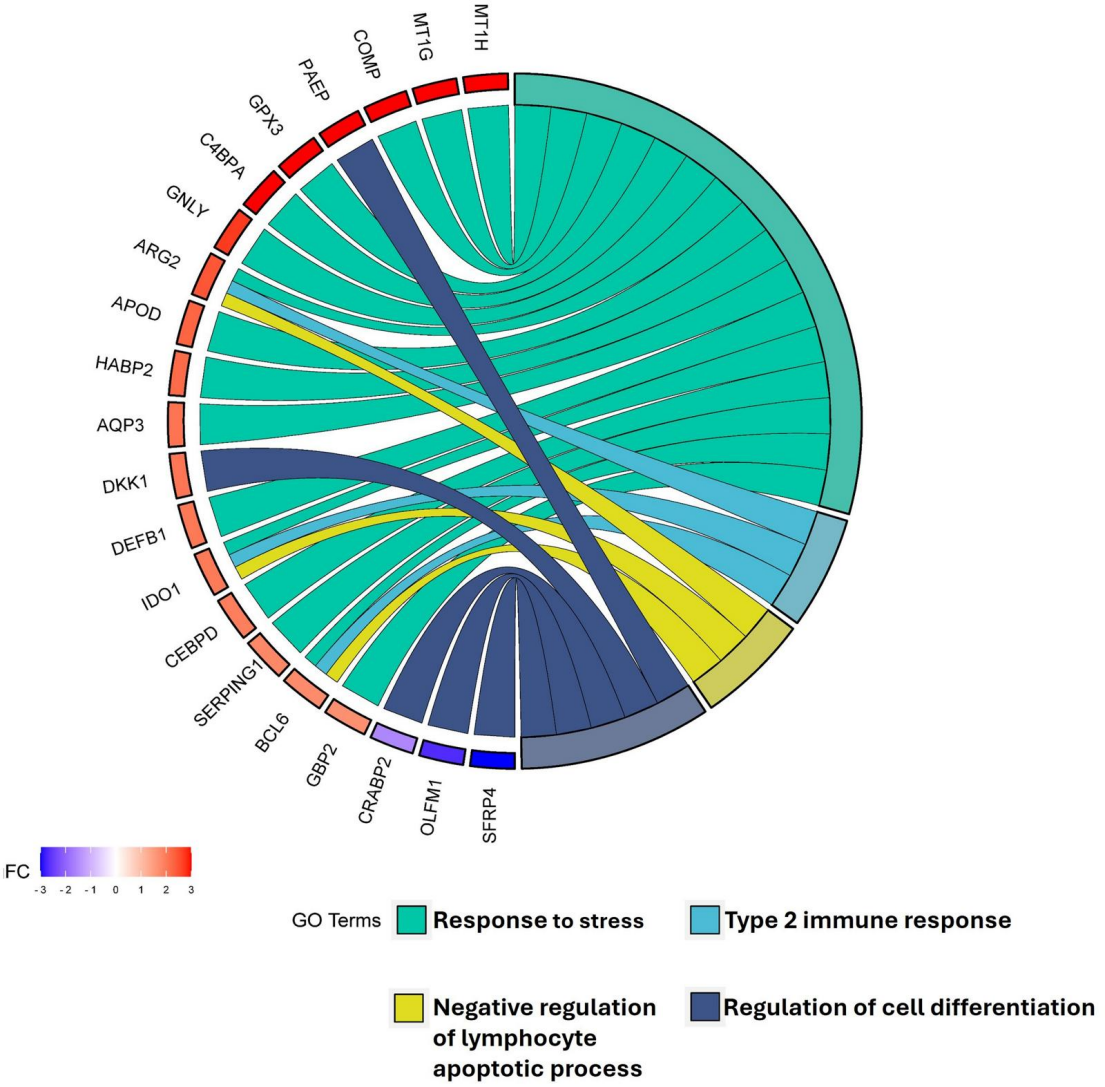
**Endometrial receptivity-associated genes and related biological processes that are differentially expressed in mid-secretory phase uterine fluid extracellular vesicles (UF-EVs).** Genes are presented on the left side of the chord plot with their log2 fold change (FC) indicated with color. GO, gene ontology.

We have previously reported, using ES and MS UF-EV samples, that endometrial receptivity can be assessed from UF-EV transcriptome using a panel of 68 endometrial tissue biomarkers (Meltsov *et al.*, 2023a). During the transition from ES to MS in endometrial tissue, 51 out of 68 receptivity biomarkers appeared as DEGs, whereas in UF-EVs there were 23 receptivity-associated DEGs, including *PAEP*, *GPX3*, *MAOA*, *DKK1*, *SERPIN1G*, and *C4BPA* which are frequently reported as DEGs in endometrial tissue during WOI. Interestingly, out of the 23 DEGs in UF-EVs, 21 are associated with biological processes, namely response to stress, type 2 immune response, negative regulation of lymphocyte apoptotic process, and regulation of cell differentiation (Fig. 5), whereas no significant enrichment of any biological process was found in the remaining 47 receptivity biomarkers.

### Changes in UF-EV and EB microRNA expression during the transitions of the endometrium

**Figure 6.**
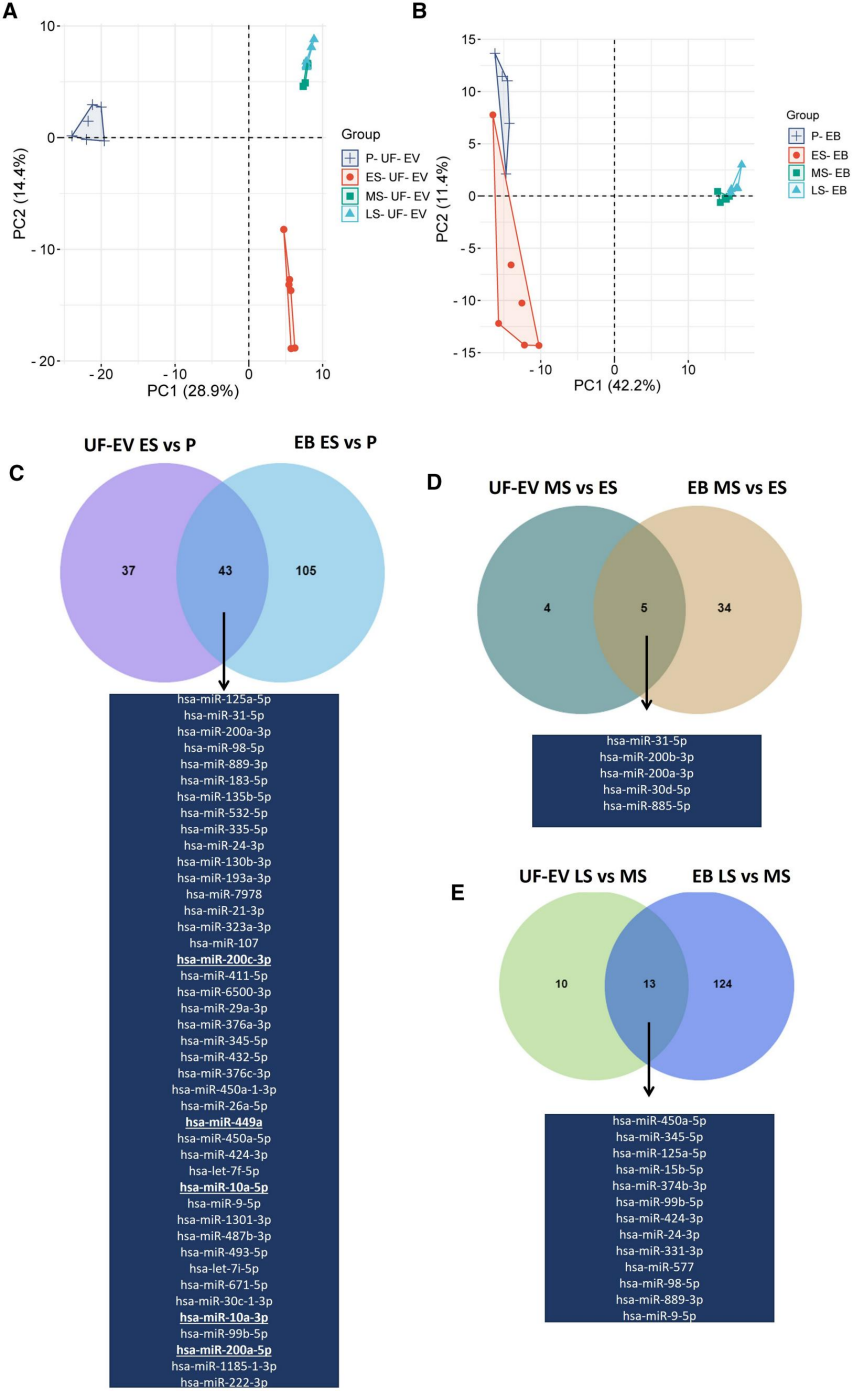
**miRNome changes in uterine fluid extracellular vesicles (UF-EV) endometrial and tissue biopsy (EB) across the menstrual cycle.** miRNome principal component analysis plot of the (**A**) UF-EV and (**B**) EB samples. Venn diagrams of differentially expressed miRNAs in UF-EV and EB in (**C**) ES compared to P phase, (**D**) MS compared to ES phase, and (**E**) LS compared to MS phase. Shared differentially expressed miRNAs are listed below diagram. miRNAs that are differentially expressed in the opposite direction in UF-EV compared to EB are in bold and underlined. P, proliferative; ES, early-secretory; MS, mid-secretory; LS, late-secretory phases.

The miRNA changes across the menstrual cycle were evaluated by small RNA sequencing of UF-EVs and paired EBs (P, n = 5 + 5; ES, n = 6 + 6; MS, n = 5 + 5; and LS, n = 5 + 5). The principal component analysis (PCA) revealed a similar clustering pattern for the UF-EVs and EB samples for each phase of the menstrual cycle, where MS and LS phases clustered closely (Fig. 6A and B). Next, DEmiR analysis was performed on consecutive phases. The most dramatic change of the miRNome of UF-EVs and EB occurred during the transition from the P to the ES phase, with 80 and 148 DEmiRs, respectively (for all comparisons, see Fig. 6C–E, Supplementary Tables S3 and S4). In each comparison, approximately half of the DEmiRs in UF-EVs were common with endometrial tissue (Fig. 6C–E). Both in UF-EVs and EBs, we observed DEmiRs previously associated with endometrial receptivity: miR-885-5p, miR-31-5p, miR-200b-3p, miR-200a-3p, and miR-30d-5p. Interestingly, the miR-200 family comprising miR-200a, miR-200b, miR-429, miR-200c, and miR-141 were found to be DE throughout all phases of the menstrual cycle in both EBs and UF-EVs, with at least one of its members being DE in each phase (Supplementary Table S5).

### miRNAs within UF-EVs are predicted to regulate gene expression in the endometrium and embryo trophectoderm lineage

**Figure 7.**
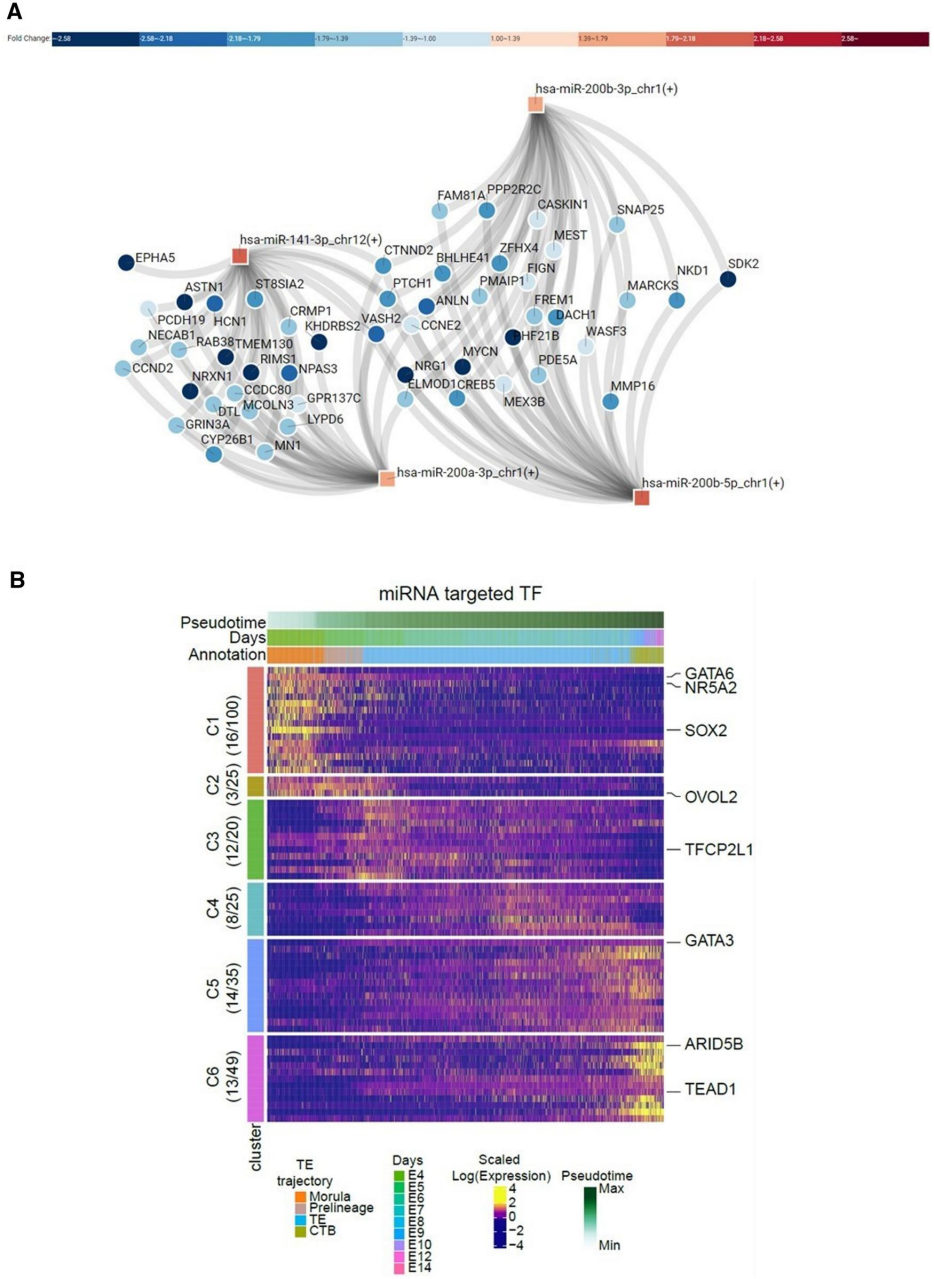
**miRNA–mRNA interactions between uterine fluid extracellular vesicles (UF-EVs), endometrium, and embryo.** (**A**) DE miRNAs in the MS phase from UF-EVs targeting DE mRNAs in the MS phase of the endometrium. (**B**) Heatmap showing the inferred expression levels of embryonal TFs targeted by DE miRNAs of MS phase UF-EVs. TFs are divided into clusters C1–C6 based on expression patterns along TE embryonic trajectory; numbers on the left indicate the number of targeted TF genes by DE miRNAs in each cluster. DE, differentially expressed; MS, mid-secretory; TF, transcription factor; TE, trophectoderm; CTB, cytotrophoblast; C, cluster; E, embryonic.

Given that only half of the DEmiRs are shared between tissue and UF-EVs, we assume that the EV-specific miRNome could play a role in intercellular communication and subsequent changes in the endometrium. To investigate the possible cell-to-cell communication further, we examined the miRNA–mRNA interactions between UF-EVs and EBs during the same menstrual cycle phase, focusing on the EV-associated DEmiRs and their predicted DEG targets within endometrial tissue. The predicted number of miRNA–mRNA interactions is 93 for the P to ES transition (Supplementary Fig. S3A), 103 for the ES to MS transition (Fig. 7A), and 15 for the MS to LS transition (Supplementary Fig. S3B). Notably, during the MS phase, three out of four miRNAs implicated in the interactions belonged to the miR-200 family. These miR-200 family members shared a significant proportion of their predicted target mRNAs (Fig. 7A).

Next, to assess the uterine–embryo interaction mediated by UF-EVs, we took the nine DEmiRs identified in the UF-EVs in the MS phase and predicted their targets among the TFs expressed in TE lineage of human embryogenesis, from our recent publication (Zhao *et al.*, 2025). We selected only the TE lineage from embryos, as it is directly exposed to UF-EVs. The analysis highlighted six miRNAs that targeted 66 TFs categorized into six distinct gene clusters (C1–C6), based on their expression profiles during TE development (Fig. 7B, Supplementary Table S6). Among these, key TFs essential for human early embryogenesis and particularly TE lineage functioning, like *GATA3*, *GATA6*, *SOX2*, *NR5A2*, and *TEAD1* were predicted as targets of the identified miRNAs from UF-EVs (Fig. 7B).

## Discussion

In this study, we established a workflow allowing multi-omics characterization from a single UF-EV sample. By exploring the surface proteome and miRNome of UF-EVs and EEO-EVs, we provide evidence that the EVs in UF originate from several cell types not restricted to the endometrial epithelium. Furthermore, by profiling the transcriptome and miRNome of paired EB and UF-EV samples, we found a resemblance in the molecular landscape of the tissue and EVs during consecutive transitions of four menstrual phases: P, ES, MS and LS phases. However, UF-EV-based receptivity testing might not only benefit from, but actually require, combining mRNA and miRNA biomarkers, as transition from MS to LS introduces modest changes in the UF-EV transcriptome, and adding miRNA biomarkers to the analytical panel could improve differentiation of the MS from the LS phase.

For the first time, we describe the UF-EV surface proteome across the menstrual cycle, which can indicate the cellular origin of EVs (Hallal *et al*., 2022). Our data revealed an enrichment of commonly used EV markers, CD9, CD63, and CD81, during the MS phase. These results suggest an increased secretion of EVs during the crucial WOI at MS, when the endometrium prepares for embryo implantation. Previous studies of CD63 expression in endometrial tissue also showed increase during the MS phase (Ng *et al.*, 2013; Vilella *et al.*, 2015). Additionally, we observed a significant increase in CD56-positive EVs during the ES and MS phases, which is in agreement with the increase of the uNK cells in the endometrium during the secretory phase (Flynn *et al.*, 2000; Biswas Shivhare *et al.*, 2015; Marečková *et al.*, 2024). On the other hand, the increase in CD3-positive EVs does not correlate with data from tissue, where a decrease in total T-cell numbers during MS has been documented (Vallvé-Juanico *et al.*, 2019). This can be explained by the fact that higher signals can indicate an increase of EV secretion by these cells or the increase of numbers of proteins on EVs and not changes in cell numbers. Indeed, the EV secretion rate can be changed without changes in cell numbers (von Lersner *et al.*, 2024). Along with CD3 and CD56, two other T-cell markers, CD4 and CD8, were detected. Previous studies on UF have identified circulating immune cells, including T cells, NK cells, and neutrophils, explaining our EV flow cytometry results (Herrera *et al.*, 2023; Strbo *et al.*, 2024). We observed that CD142, or tissue factor, which is a marker associated with coagulation, is increased the most in UF-EVs in the MS phase, consistent with previously reported EB staining (Altmäe *et al.*, 2011). During the P phase, CD142 expression is low (Krikun *et al.*, 2009), aligning with our observations of minimal CD142 marker expression on UF-EVs during this phase. Furthermore, endothelial cell markers (CD105, CD31, and CD146) were also identified on UF-EVs. These endothelial markers are less likely to come from biopsy-caused injury but rather they originate from EV movement via transcytosis, as has been described for other tissues (Serra and Sundaram, 2021; Ramos-Zaldívar *et al.*, 2022; Iannotta *et al.*, 2024). Moreover, we profiled the surface proteome of EEO-EVs as a reference of endometrial epithelial cell EVs. It is worth noting that the EEO model we are using is of the apical-in type, which, in principle, represents EVs released into the endometrial stroma and vasculature (Turco *et al.*, 2017). However, in a metabolome study, researchers isolated intra-organoid fluid (IOF) and extra-organoid fluid (EOF) and found that 50% of the metabolites were shared between them, with 40% being exclusive to EOF and 10% to IOF (Simintiras *et al.*, 2021). This finding might not directly apply to EVs, as their movement is not uni-directional; instead, they exhibit bi-directional secretion, possibly involving mechanisms like transcytosis. Still, we observed consistent expression of CD29, CD133, CD326/EpCAM, CD24, CD44, CD142/Tissue factor, CD146, HLA-DR/DP/DQ, ROR1, and SSEA-4 in both UF-EVs and EEO-EVs, which indicates that these are the surface protein markers expressed by the endometrial epithelium. Furthermore, we observed that the miRNome of UF-EVs is much more complex than the miRNome of EEO-EVs. Collectively, these findings indicate that UF-EVs originate from several cell types, including epithelial, endothelial, and immune cells.

Transcriptomic analysis across the menstrual cycle in EB indicated that the biggest changes occur during transition from ES to MS, consistent with another study where global transcriptomic derepression was detected in WOI (Sebastian-Leon *et al.*, 2021). However, in the UF-EV transcriptome, the biggest change occurred during the transition from the P to the ES phase (>5500 DEGs), where most of the DEGs were downregulated and surprisingly associated with sensory and stimulus detection. This could be explained by a bigger proportion of plasma exudate in UF during the P phase. Indeed, studies in rodents have shown that estrogen can induce vascular permeability and alter the epithelial tight junctions in the endometrium during the P phase (McRae, 1988). Nonetheless, by focusing on the gene expression of two big protein families (histones and metallothioneins) across the menstrual cycle, we saw a correlation between expression in UF-EVs and paired EB samples even in the P phase. Interestingly, during the P and ES phases in both tissue and UF-EV samples, we observed higher expression of histones, which corresponds to the proliferation and division of cells during these phases of the menstrual cycle. In contrast, the MS phase showed decreased expression, aligning with the expected halt in cellular proliferation in the decidualizing endometrium (Wang et al., 2020). Metallothioneins are part of several receptivity biomarker panels (Díaz-Gimeno *et al.*, 2011; Meltsov *et al.*, 2023b), whereas histones are not, likely because replication-dependent histone mRNAs are non-polyadenylated and cannot be amplified by frequently used poly-A-dependent library preparation (Marzluff *et al.*, 2008). By utilizing a library preparation method that captures coding regions, we recently detected for the first time the dynamic expression of histone family mRNA in endometrium (Apostolov *et al.*, 2024) and now also in UF-EVs.

We have previously assessed endometrial receptivity based on UF-EV transcriptome with 100% specificity and 75% sensitivity utilizing 68 tissue-based biomarkers (Meltsov *et al.*, 2023a). Although there is low concordance between different endometrial transcriptomic studies (Walker *et al.*, 2023), we identified in our EB data, 51 DEGs out of 68 receptivity-associated biomarkers which have been reported in a previous meta-analysis (Altmäe *et al.*, 2017; Meltsov *et al.*, 2023b). In our UF-EV sample dataset, only 23 out of 68 genes appeared as differentially expressed, implying that UF-EV-based receptivity test could have improved performance by extending tissue-based biomarkers with common UF-EV and EB DEGs.

By profiling the miRNome of UF-EVs and paired EB samples, we observed that half of the DEmiRs in each phase of the menstrual cycle were shared between the tissue and EVs. Among the shared miRNAs, all of them had the same direction in expression change in both EVs and EBs, except for four miRNAs during the transition from P to ES phase, suggesting specific regulation of miRNA expression in EVs, resembling the endometrial tissue across the menstrual cycle. Among the shared miRNAs in the MS phase, miR-200b-3p, miR-200a-3p, miR-30d, miR-31-5p, and miR-885 all had been previously associated with a WOI signature in EB or UF (Kuokkanen *et al.*, 2010; Altmäe *et al.*, 2013; Kresowik *et al.*, 2014; Vilella *et al.*, 2015; Sigurgeirsson *et al.*, 2017; Rekker *et al.*, 2018). miR-200b-3p has also been found to be upregulated in UF-EVs samples right before embryo transfer from subfertile patients who achieved pregnancy in that cycle (Ibañez-Perez *et al.*, 2022). Moreover, it has been shown that supplementing mice embryos with miR-30d increases their adhesion potential (Vilella *et al.*, 2015), indicating its importance in maternal–embryo communication. Throughout the menstrual cycle, we observed constant differential expression of miRNAs from the miR-200 family, and in the MS phase, their elevated expression correlated with downregulation of 48 putative target genes. Collectively, these data uphold that the miR-200 family has an important role in endometrial preparation for implantation that also has diagnostic and therapeutic potential.

A recent study described the transcriptional changes in human blastocysts exposed to EVs secreted by human primary uterine epithelial cells (Segura-Benítez *et al.*, 2025). However, it is important to note that UF-EVs are more complex in composition than the endometrial epithelial EVs used for blastocyst treatment in this recent study. Therefore, the approach taken by the current study and exploring the UF-EVs, as the environment for human embryos, provides a more comprehensive way to highlight the putative uterine and embryonal molecular interactions. In the current study, we predicted the uterine–embryo interactions using our recently published comprehensive human embryo reference tool of single-cell RNA-sequencing data (Zhao *et al.*, 2025). We found that UF-EV-associated miRNAs may play an important role in human early embryogenesis, as important TFs involved in TE lineage functioning were identified as the pivotal targets for MS UF-EV miRNAs. The key TFs targeted by the UF-EV miRNAs include GATA6, involved in primitive endoderm differentiation (Schrode *et al.*, 2014); GATA3, regulating TE lineage commitment and the morula-to-blastocyst transition (Home *et al.*, 2009); TEAD1, which is essential for inner cell mass segregation (Regin *et al.*, 2023); SOX2, important for TE formation (Keramari *et al.*, 2010); and NR5A2, crucial for the first lineage segregation in embryogenesis (Lai *et al.*, 2023). We also identified three TFs, ARID5B, TFCP2L1, and OVOL2, that were downregulated in blastocysts exposed to epithelial cell-derived EVs (Segura-Benítez *et al.*, 2025) and were also predicted as target genes of the UF-EV miRNAs in the current study. Our study also revealed that miR-141-3p, miR-200a-3p, and miR-200b-3p found in MS UF-EVs modulate mRNA expression in both endometrial tissue and TE lineage of human embryogenesis. This finding suggests that these miRNAs may play a central role in orchestrating endometrial receptivity and embryo implantation, and the molecular mechanisms of these miRNAs on target cells require deeper analysis.

In summary, we provide evidence that UF-EVs reflect dynamic changes in endometrial tissue throughout the menstrual cycle both on mRNA and miRNA levels. As several immune cell type markers, like CD3 and CD56, were detected on UF-EVs on the protein level, it is plausible that UF-EVs could also be used for profiling the immunological status of the endometrium. Combining biomarkers from both omics levels, as well as of mRNAs and miRNAs, is probably a necessity to assure differentiation between MS and its preceding and succeeding phases. This implementation would be beneficial for the development of a minimally invasive, multi-omics endometrial receptivity testing that reflects the full complexity of endometrial environment. However, it is important to acknowledge that the present study is limited by the relatively small sample size, which may introduce subtle bias in the detected omics profiles. Moreover, the absence of universal and standardized procedure for collecting UF samples in gynecological practice may challenge the reproducibility of our results.

## Supplementary Material

hoaf010_Supplementary_Data

## Data Availability

The data are not publicly available due to ongoing studies. Please contact the corresponding author for access to the data.
