# Supplementary figures and images for: Multi-omics analysis of uterine fluid extracellular vesicles reveals a resemblance with endometrial tissue across the menstrual cycle: biological and translational insights
Source: Hum Reprod Open. 2025 Feb 24;2025(2):hoaf010. doi: 10.1093/hropen/hoaf010 (PMC11904304; doi:10.1093/hropen/hoaf010)

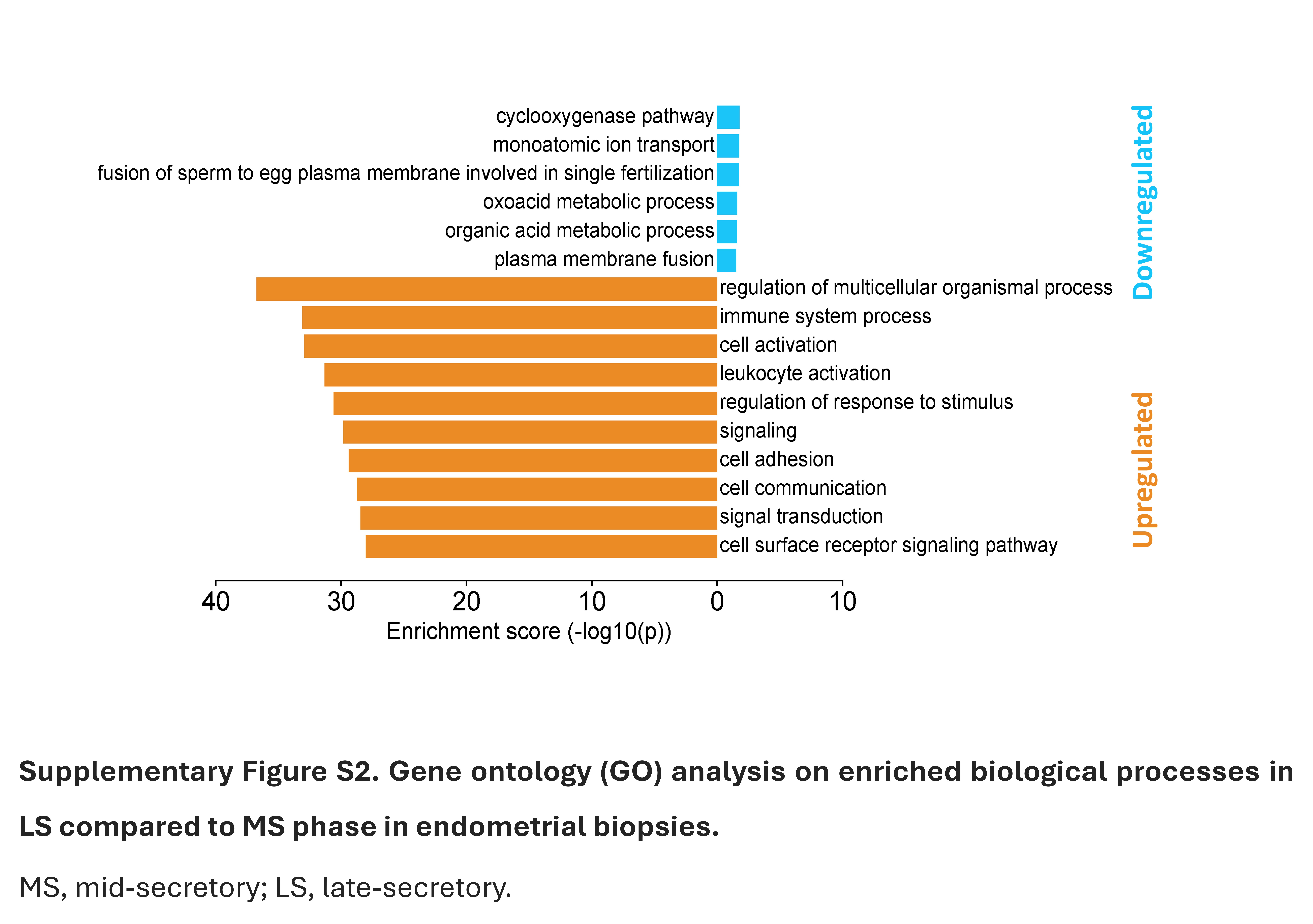

Supplement: hoaf010_Supplementary_Data [file hoaf010_supplementary_data.zip › Supplementary Figure S2.jpg]
